# Optimized protocol for naive human pluripotent stem cell-derived trophoblast induction

**DOI:** 10.1016/j.xpro.2021.100921

**Published:** 2021-10-29

**Authors:** Shingo Io, Yoshiki Iemura, Yasuhiro Takashima

**Affiliations:** 1 Department of Life Science Frontiers, CiRA, Kyoto University, Kyoto 606-8507, Japan; 2 Department of Gynecology and Obstetrics, Kyoto University Graduate School of Medicine, Kyoto 606-8507, Japan; 3 Department of Obstetrics and Gynecology, Kosaka Women’s Hospital, Osaka 577-0807, Japan; 4 Optional Department of Diagnostic Pathology, Kyoto University Graduate School of Medicine, Kyoto 606-8507, Japan; 5 Japan Society for the Promotion of Science, Tokyo 102-0083, Japan

**Keywords:** Cell Biology, Cell culture, Cell Differentiation, Developmental biology, Flow Cytometry/Mass Cytometry, Molecular Biology, Stem Cells

## Abstract

Human trophoblasts arise from the morula as trophectoderm, which differentiates into cytotrophoblast, syncytiotrophoblast, and extravillous trophoblast after implantation. Here, we present a robust step-by-step protocol to induce trophectoderm (TE) from naive human pluripotent stem cells (PSCs) corresponding to pre-implantation epiblast. Our culture system (TE induction and ACE condition) mimics the entire trophoblast development including the molecular events.

For complete details on the use and execution of this protocol, please refer to Io et al. (2021).

## Highlights

• Direct induction to human trophectoderm from naive PSCs
• Naive PSC-derived trophectoderm can be isolated by flow cytometry
• Naive PSC-derived trophectoderm differentiates to cytotrophoblast in ACE condition
• Cytotrophoblast stem cells can be established from chorionic villi in ACE condition

## Graphical abstract

**Figure:**
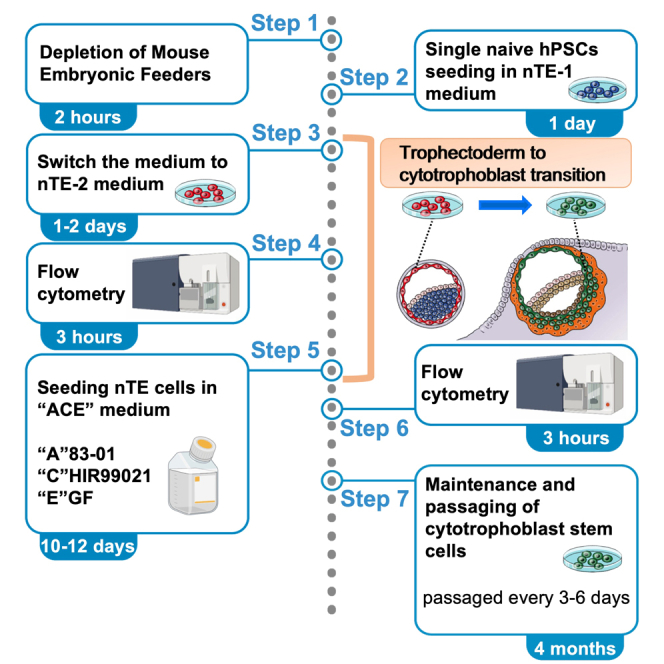

## Before you begin

The protocol below describes the steps for using a specific human embryonic stem cell line (H9). We have also used this protocol on human induced pluripotent stem cell lines (409B2 and AdiPS1). For simplicity, 6-well plates are usually used. All cells are cultured in 5% O_2_, 5% CO_2_ unless noted otherwise. Passages and medium change are performed in normoxia.

### Preparation of stock solutions for trophoblast derivation and culture

**Timing: 3 h**

1. Prepare aliquots of stock solutions to avoid freeze-thaw cycles.

**Table:** Storage and working concentrations of reagents

Reagent	Storage concentration	Working concentration	Storage conditions
Trypsin	2.5%	0.025%	Store at −20°C (for long-term storage)
Collagenase IV	10 mg/mL	1 mg/mL	
Knockout Serum Replacement (KSR)	Directly aliquoted	1:5	
MEM non-essential amino acids (NEAA)	Directly aliquoted	1:100	Store at 4°C
2-Mercaptoethanol (2ME)	100 mM	0.1 mM	
7.5w/v% Albumin D-PBS (-) Solution, from Bovine Serum (BSA)	Directly aliquoted	1:750	
Recombinant human basic fibroblast growth factor (bFGF)	10 μg/mL	4 ng/mL	Store at −80°C (for long-term storage)
Recombinant human BMP4 protein	100 μg/mL	10 ng/mL	
Recombinant human EGF protein	100 μg/mL	50 ng/mL	
Recombinant human LIF	1 μg/mL	10 ng/mL	
CHIR99021	10 mM	1 or 2 μM	Store at −80°C
PD0325901	10 mM	1 or 2 μM	
Gö6983	5 mM	2 μM	
Y-27632	10 mM	10 μM	
A83-01	2.5 mM	1, 2 or 7.5 μM	
JAK inhibitor I	5 mg/mL	1 μg/mL	
XAV939	20 mM	2 μM	
Valproic acid sodium salt (VPA)	0.3 M	1 mM	
NRG1	100 μg/mL	100 ng/mL	
Forskolin	100 mM	2 μM

2. Prepare 10 mL aliquots of Trypsin, KSR, NEAA and 7.5% BSA.
3. Resuspend and filter Collagenase IV in PBS to a final concentration of 10 mg/mL and aliquot.
4. Resuspend 70 μL of 2ME in 10 mL of PBS to a final concentration of 100 mM. Aliquot in a volume of 500 μL.

***Note:*** Hazardous. Avoid breathing fumes. Wear glasses, gloves, and other appropriate protection and handle with care.

5. Resuspend 100 μg of bFGF in 10 mL of PBS with 0.01% BSA to a final concentration of 10 μg/mL. Aliquot in a volume of 100 μL. Store the aliquots at −80°C for ≤6 months. After thawing an aliquot, store it at 4°C for ≤2 weeks.
6. Resuspend 10 μg of BMP4 in 100 μL of 4 mM HCl with 0.1% BSA to a final concentration of 100 μg/mL. Aliquot in a volume of 10 μL. Store the aliquots at −80°C for ≤6 months. After thawing an aliquot, store it at 4°C for ≤2 weeks.
7. Resuspend 200 μg of EGF liquid in 1.8 mL of PBS with 0.1% BSA to a final concentration of 100 μg/mL. Aliquot in a volume of 100 μL. Store the aliquots at −80°C for ≤6 months. After thawing an aliquot, store it at −20°C for ≤2 weeks.
8. Resuspend 1 mg of LIF in 100 mL of PBS with 0.1% BSA to a final concentration of 1 μg/mL and aliquot in a volume of 100 μL. Store the aliquots at −80°C for ≤6 months. The LIF solution should not be stored at 4°C for more than one week.
9. Resuspend 25 mg of CHIR99021 in 5,373 μL of DMSO to a final concentration of 10 mM. Aliquot in a volume of 20 μL. Store the solution at −80°C for ≤6 months and avoid repeated freeze-thaw cycles. The CHIR99021 solution should not be stored at 4°C for more than one week.

***Note:*** Toxic. Avoid contact and inhalation, Wear a mask and gloves.

10. Resuspend 50 mg of PD0325901 in 10,359 μL of DMSO to a final concentration of 10 mM. Aliquot in a volume of 20 μL. Store the solution at −80°C for ≤6 months. The PD0325901 solution should not be stored at 4°C for more than one week.
11. Resuspend 10 mg of Gö6983 in 4,520 μL of DMSO to a final concentration of 5 mM. Aliquot in a volume of 50 μL. Store the solution at −80°C for ≤6 months. The Gö 6983 solution should not be stored at 4°C for more than one week.
12. Resuspend 50 mg of Y-27632 in 14.7 mL of distilled water (DW) to a final concentration of 10 mM. Aliquot in a volume of 100 μL. Store the aliquots at −80°C for ≤6 months. After thawing an aliquot, store it at 4°C for ≤2 weeks.
13. Resuspend 50 mg of A83-01 in 4,740 μL of DMSO to a final concentration of 2.5 mM. Aliquot in a volume of 50 μL. Store the aliquots at −80°C for ≤6 months. The A83-01 solution should not be stored at 4°C for more than one week.
14. Resuspend 5 mg of JAK inhibitor I in 323 μL of DMSO to a final concentration of 10 mM. Aliquot in a volume of 30 μL. Store the solution below −80°C for ≤6 months and avoid repeated freeze-thaw cycles.
15. Resuspend 10 mg of XAV939 in 1.6 mL of DMSO to a final concentration of 20 mM. Aliquot in a volume of 20 μL. Store the aliquots at −80°C for ≤6 months. The XAV-939 solution should not be stored at 4°C for more than one week.
16. Resuspend and filter 25 g of valproic acid sodium salt (VPA) in 501 mL of DW to a final concentration of 0.3 M. Aliquot in a volume of 30 μL.
17. Resuspend and filter 50 μg of NRG1 in 500 μL of 20 mM citrate (pH3.0) to a final concentration of 100 μg/mL. Aliquot in a volume of 20 μL.
18. Resuspend 10 mg of Forskolin in 244 μL of DMSO to a final concentration of 100 mM. Aliquot in a volume of 20 μL.

### Preparation of mouse embryonic fibroblast (MEF) feeder plates

**Timing: 1–2 weeks**

19. Thawing MEF feeder cells
  a. Prepare 10 cm dishes and the MEF culture medium.
  b. Add 4 mL of 0.1% gelatin solution to each dish and incubate for 10 min at room temperature.
  c. Remove the cryovial containing MEF from liquid nitrogen and thaw for 1–2 min in a 37°C water bath.
  d. Transfer the MEF in the cryovial to a 15-mL tube and add 5 mL of MEF culture medium.
  e. Centrifuge at 1,300 rpm (330 *g*) for 3 min, aspirate the supernatant, and resuspend the pellet with MEF culture medium at a density of 5.0 × 10^4^ cells/mL.
  f. Aspirate the gelatin solution from the well and add 10 mL of MEF suspension to each dish.
20. Passage of MEF feeder cells
  a. Incubate the MEF feeder dishes at 37°C until the culture cells reach subconfluence.
  b. Wash the dishes with PBS 2 times.
  c. Add 1 mL of trypsin and incubate at 37°C for 5–10 min.
  d. Transfer the dissociated cells into a 50-mL tube and add MEF culture medium at 5 times the amount.
  e. Centrifuge at 1,300 rpm for 3 min, aspirate the supernatant, and resuspend the pellet with MEF culture medium.
  f. Seed the cells on newly prepared gelatin-coated 10 cm dishes at a 1:4-1:6 split ratio.
21. Inactivate the MEF feeder cells.
  a. Harvest the MEF feeder cells with trypsin after the cells reach subconfluence.
  b. Resuspend the dissociated cells with MEF culture medium at a density of 1.0–5.0 × 10^6^ cells/mL.
  c. Irradiate the cells at 3,285 cGy/min for 40 min with a GAMMACELL 40 EXACTOR.
  d. Centrifuge at 1,300 rpm for 3 min, aspirate the supernatant, and resuspend the pellet with CELLBANKER 1 (ZENOGEN PHAMA) at a density of 2.0 × 10^6^ cells/mL.
  e. Aliquot in a volume of 500 μL into cryovials.
  f. Cryopreserve the cells in liquid nitrogen.
22. Thawing the inactivated MEF feeder cells for use in PSC culture.
  a. Thaw MEF in the cryovial for 1–2 min in a 37°C water bath.
  b. Transfer the content of the cryovial to a 15-mL tube and then add 5 mL of MEF culture medium.
  c. Centrifuge at 1,300 rpm for 3 min, aspirate the supernatant, and resuspend the pellet with MEF culture medium.
  d. Seed the cells to a gelatin-coated 6-well plate (1.0 × 10^6^ cells/plate).
  e. Incubate the plate overnight at 37°C.

***Note:*** We usually establish MEF feeder cells from embryonic day 13.5 Jcl:ICR mouse embryos.

***Note:*** MEF feeder cells can be expanded more in 5% oxygen than in 20% oxygen. The expanding MEF feeder cells should be used within 10 passages.

***Note:*** The MEF feeder cells can be also inactivated with Mitomycin C (MMC) treatment. When you use MMC, add the MMC-containing medium at a density of 10 μg/mL and incubate for 2–3 hours before harvesting the cells.

***Note:*** Prepared dishes with the inactivated MEF layer should be used within 1 week.

**Table:** MEF culture medium

Reagent	Final concentration	Amount
DMEM/Ham’s F-12 (High Glucose)	n/a	∼45 mL
2ME	0.1 mM	50 μL
FBS	10%	5 mL
**Total**	**n/a**	**50 mL**

***Note:*** Store the solution at 4°C for up to 8 weeks.

### Maintenance of human primed PSCs

**Timing: 1–2 weeks**

23. Prepare an inactivated MEF plate the day before step 24.
24. Thawing human primed PSCs
  a. Prepare an inactivated MEF-feeder plate and primed PSC medium.
  b. Warm the primed PSC medium in a 37°C water bath.
  c. Remove the cryovial of primed PSCs from liquid nitrogen and thaw 1–2 min in the 37°C water bath.
  d. Transfer the contents of the cryovial to a 15-mL tube and then add 5 mL of primed PSC medium.
  e. Centrifuge at 1,300 rpm for 3 min, aspirate the supernatant, and resuspend the pellet with 2 mL of primed PSC medium.
  f. Aspirate the MEF culture medium from the inactivated MEF-feeder plate and wash the wells with 1 mL of PBS 2 times.
  g. Add 2 mL of primed PSCs suspension to the well.
25. Replenish the medium every day until the culture cells reach 70–80% confluence.
26. The primed PSCs are passaged on the newly prepared MEF plates as clumps after treatment with CTK solution for 15–30 min.

***Note:*** If you use a cryopreservation reagent that has a strong cytotoxic effect, such as DAP213, you should add prewarmed medium to the cryovials and thaw quickly.

**Table:** Primed PSC medium

Reagent	Final concentration	Amount
DMEM/Ham’s F-12	n/a	∼40 mL
NEAA	1**×**	500 μL
KSR	20%	10 mL
2ME	0.1 mM	50 μL
bFGF	4 ng/mL	20 μL
**Total**	**n/a**	**50 mL**

***Note:*** Store the solution at 4°C for up to one week.

**Table:** CTK solution

Reagent	Final concentration	Amount
PBS	n/a	39 mL
2.5 g/l-Trypsin/1 mmol/l-EDTA Solution, with Phenol Red	0.025%	500 μL
Collagenase IV	1 mg/mL	50 mg
KSR	20%	10 mL
0.1 mol/l-Calcium Chloride Solution	1 mM	500 μL
**Total**	**n/a**	**50 mL**

***Note:*** Aliquot and freeze the solution at −20°C or less.

### Chemical conversion to naive PSCs

The chemical conversion to naive PSCs is performed as previously described (Guo et al., 2017). The 5i/L/A and NK2 transgene methods can also be used for primed-to-naive resetting (Theunissen et al., 2014; Takashima et al., 2014).

**Timing: 2–3 weeks**

**Figure 1:**
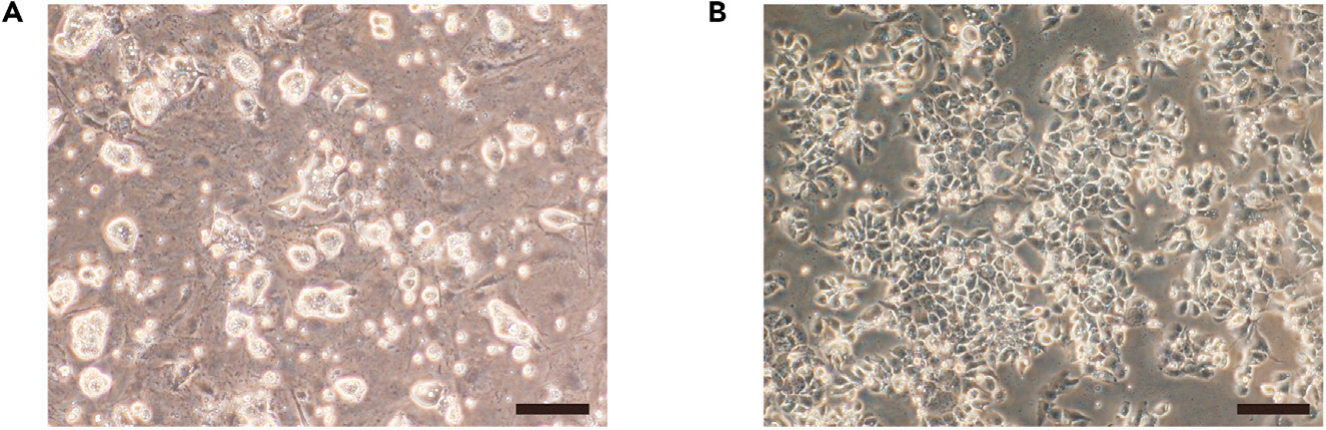
Trophectoderm-like cells derived from human naive pluripotent stem cells (A) Phase contrast image of human naive pluripotent stem cells. (B) Phase contrast image of trophectoderm-like cells on day 3 of the differentiation. Scale bars, 100 μm.
34. After the medium is changed to t2iLGö medium, the cells can be dissociated with Accutase and passaged on newly prepared MEF-coated plates at a 1:4 split ratio every 3–5 days.

***Note:*** Naive PSCs should be passaged every three to five days. Over-confluency affects cell division and may reduce the efficiency of subsequent naive PSC-derived trophectoderm (nTE) and naive PSC-derived cytotrophoblasts (nCTs) differentiation.

***Note:*** Mycoplasma contamination is also possible should the cells not grow. Mycoplasma contamination can be checked using a MycoAlert Mycoplasma Detection Kit.

27. Prepare an inactivated MEF plate the day before step 28.
28. Single-cell collection of primed PSCs.
  a. Incubate the primed PSCs on MEF feeder cells in primed PSC medium with 10 μM Y-27632 for more than one hour.
  b. Aspirate the medium of the well and wash the well with 1 mL of PBS 2 times.
  c. Add 500 μL TrypLE Express and incubate in a humidified incubator at 37°C for 5 min.
  d. Transfer the dissociated cells to a 15-mL tube and add 5 mL of Wash medium.
  e. Centrifuge at 1,300 rpm for 3 min, aspirate the supernatant, and resuspend the pellet with 1 mL primed PSC medium with 10 μM Y-27632.
  f. Prepare a 6-well plate and add 1 mL of 0.1% gelatin solution to a well.
  g. Aspirate the gelatin solution after 10 min of incubation at room temperature and transfer the cell suspension in 1 mL primed PSC medium with 10 μM Y-27632 on the gelatin-coated well.
  h. Incubate at 37°C for 2 h to make the MEF feeder cells attach onto the well.
  i. Collect the medium with floating PSCs and count the number of cells.
29. Seed the PSCs at a density of 1 × 10^4^ cells/cm^2^ on inactivated MEF feeder cells in primed PSC medium with 10 μM Y-27632.
30. On the next day, switch the medium to cRM-1 medium.
31. On day 3, replace the medium with cRM-2 medium.
32. Dome-shaped naive PSC colonies are observed around two weeks after plating.
33. Cells are split every 5–7 days after dissociation with Accutase. Fully reset naive PSCs are passaged and maintained on MEF feeders in t2iLGö medium (Figure 1A).

**Table:** Wash medium

Reagent	Final concentration	Amount
DMEM/Ham’s F-12	n/a	∼50 mL
7.5% BSA	0.1%	667 μL
**Total**	**n/a**	**50 mL**

***Note:*** Store the solution at 4°C for up to 8 weeks.

**Table:** cRM-1 medium

Reagent	Final concentration	Amount
NDiff227	n/a	∼10 mL
PD0325901	1 μM	1 μL
LIF	1 ng/mL	10 μL
VPA	1 mM	33 μL
**Total**	**n/a**	**10 mL**

***Note:*** NDiff227 is a defined, serum-free, N2- and B-27 supplemented medium sold by Takara Bio. NDiff227 is also known as N2B27 medium (Takashima et al., 2014).

***Note:*** Store the solution at 4°C for up to one week.

**Table:** cRM-2 medium

Reagent	Final concentration	Amount
NDiff227	n/a	∼50 mL
PD0325901	1 μM	5 μL
LIF	1 ng/mL	50 μL
Gö6983	2 μM	20 μL
XAV939	2 μM	5 μL
**Total**	**n/a**	**50 mL**

***Note:*** Store the solution at 4°C for up to one week.

**Table:** t2iLGö medium

Reagent	Final concentration	Amount
NDiff227	n/a	∼50 mL
PD0325901	1 μM	5 μL
LIF	1 ng/mL	50 μL
Gö6983	2 μM	20 μL
CHIR99021	1 μM	5 μL
**Total**	**n/a**	**50 mL**

***Note:*** Store the solution at 4°C for up to one week.

## Key resources table

**Table:** 

REAGENT or RESOURCE	SOURCE	IDENTIFIER
**Antibodies**		

Mouse monoclonal anti-CD249 (ENPEP), PE conjugated (clone 2D3/APA) (1:100 dilution)	BD Biosciences	Cat#564533; RRID: AB_2738838
Mouse monoclonal anti-CD249 (ENPEP), BV421 conjugated (clone 2D3/APA) (1:100 dilution)	BD Biosciences	Cat#744872; RRID: AB_2742549
Recombinant anti-TROP2 (TACSTD2), biotin conjugated (clone REA916) (1:500 dilution)	Miltenyi Biotec	Cat#130-115-054; RRID: AB_2726879
Recombinant anti-TROP2 (TACSTD2), Alexa Fluor 488 conjugated (clone 77220) (1:100 dilution)	R&D systems	Cat#FAB650G; RRID: not available
Mouse monoclonal anti-HLA-ABC, Pacific Blue conjugated (clone W6/32) (1:100 dilution)	Biolegend	Cat#311418; RRID: AB_493669
Recombinant anti-human CD327 (SIGLEC6), biotin conjugated (clone REA852) (1:100 dilution)	Miltenyi Biotec	Cat#130-112-708; RRID: AB_2725881
Rat monoclonal anti-Streptavidin, APC conjugated (1:1000 dilution)	Biolegend	Cat#405207; RRID: not available
DAPI (4′,6-Diamidino-2-phenylindole dihydrochloride) (1:1000 dilution)	Sigma-Aldrich	Cat#D9542; RRID: not available

**Chemicals, peptides, and recombinant proteins**		

iMatrix 511 silk (Laminin-E8)	Nippi, Japan	Cat#892021
Geltrex LDEV-Free, hESC-Qualified, reduced growth factor basement membrane matrix	Thermo Fisher Scientific (Invitrogen)	Cat#A1413302
Collagen IV	Corning	Cat#354233
Trypsin	Thermo Fisher Scientific (Invitrogen)	Cat#15090-046
Collagenase IV	Thermo Fisher Scientific (Invitrogen)	Cat#17104-019
0.1 mol/l-Calcium Chloride Solution	Nacalai tesque, Kyoto, Japan	Cat#16973-64
Accutase	Sigma-Aldrich	Cat#A6964
2.5 g/l-Trypsin/1 mmol/l-EDTA Solution, with Phenol Red	Nacalai tesque, Kyoto, Japan	Cat#32777-15
TrypLE Express Enzyme	Thermo Fisher Scientific	Cat#12604021
Stem-Cellbanker	ZENOGEN PHAMA	Cat#CB045
Cell Banker 1	ZENOGEN PHAMA	Cat#CB011
Penicillin-Streptomycin (10,000 U/mL)	Thermo Fisher Scientific	Cat#15140122
Deoxyribonuclease I from bovine pancreas Type IV (DNase)	Sigma-Aldrich	Cat#D5025
HBSS, 10**×**, no Calcium, no Magnesium, no Phenol Red	Thermo Fisher Scientific (Invitrogen)	Cat#14185052
Bovine Serum Albumin (BSA)	Sigma-Aldrich	Cat#A2153
Fetal bovine serum (FBS)	Thermo Fisher Scientific	Cat#10437028
Knockout Serum Replacement (KSR)	Thermo Fisher Scientific (Invitrogen)	Cat#10828028
DMEM/Ham's F-12	Nacalai tesque, Kyoto, Japan	Cat#08460-95
DMEM (High Glucose)	Nacalai tesque, Kyoto, Japan	Cat#08458-16
HEPES	Sigma-Aldrich	Cat#H3375
NDiff227	Takara Bio	Cat#Y40002
MEM non-essential amino acids (NEAA) (100**×**)	Thermo Fisher Scientific (Invitrogen)	Cat#11140-050
2-Mercaptoethanol (2ME)	Sigma-Aldrich	Cat#M3148
7.5w/v% Albumin D-PBS (-) Solution, from Bovine Serum (BSA)	Wako	Cat#012–23881
Insulin, Transferrin, Selenium, Ethanolamine Solution (ITS -X), 100**×**	Life Technologies	Cat#51500-056
Mitomycin C	Wako	Cat#139–18711
recombinant human basic fibroblast growth factor	Oriental Yeast	Cat#NIB47079000
CHIR99021	Sigma-Aldrich	Cat#SML1046
PD0325901	Tocris	Cat#4192
recombinant human LIF	Peprotech	Cat#300-05
Gö6983	Tocris	Cat#2285
Y-27632 (hydrochloride)	Cayman	Cat#10005583
A83-01	Tocris	Cat#2939
recombinant human BMP-4 protein	R&D systems	Cat#314-BP
JAK inhibitor I	Merck	Cat#420099
recombinant human EGF protein	R&D systems	Cat#236-EG
Forskolin	Wako	Cat#067–02191
Human neuregulin-1 (NRG1)	Cell Signaling	Cat#5218SC
XAV939	Selleck Chemicals	Cat#S1180
Valproic acid sodium salt	Sigma-Aldrich	Cat#P4543
RBC Lysis Buffer, 10**×**	Santa Cruz Biotechnology	Cat#sc-296258

**Critical commercial assays**		

MycoAlert Mycoplasma Detection Kit	Lonza	Cat#LT07-118

**Experimental models: Cell lines**		

Human embryonic stem cell line: H9 (WA09)	WiCell Research Institute	hPSCreg ID: WAe009-A
Human induced pluripotent stem cell line: 409B2	Okita et al., 2011	N/A
Human induced pluripotent stem cell line: AdiPS 1	University of Cambridge	hPSCreg ID: CAMi004-A

**Software and algorithms**		

FlowJo software 10.6.1	FlowJo, LCC	https://www.flowjo.com/; RRID:SCR_008520

**Others**		

Falcon 70 μm cell strainer	Corning	Cat#352350
BioLite 6 well Multidish (6-Well cell culture plates)	Thermo Fisher Scientific	Cat#130184
BioLite 12 well Multidish (12-Well cell culture plates)	Thermo Fisher Scientific	Cat#130185
BioLite 24 well Multidish (24-Well cell culture plates)	Thermo Fisher Scientific	Cat#130186
Greiner CELLSTAR 10 cm cell culture dishes	Greiner Bio-One	Cat#664160
15 mL VIOLAMO polypropylene conical tubes	AS ONE	Cat#VIO-15BN
50 mL VIOLAMO polypropylene conical tubes	AS ONE	Cat#VIO-50BN
Cryovials	Sarstedt K.K.	Cat#72.694.006
FSX100 Inverted Microscope	OLYMPUS	N/A
FACSAria III cell sorter	BD Biosciences	N/A

## Materials and equipment

**Table:** nTE-1 medium

Reagent	Final concentration	Amount
NDiff227	n/a	∼50 mL
A83-01	2 μM	40 μL
PD0325901	2 μM	10 μL
BMP4	10 ng/mL	5 μL
**Total**	**n/a**	**50 mL**

***Optional:*** BMP4 can be removed from nTE-1 medium.

***Note:*** Store the solution at 4°C for up to one week.

**Table:** nTE-2 medium

Reagent	Final concentration	Amount
NDiff227	n/a	∼50 mL
A83-01	2 μM	40 μL
PD0325901	2 μM	10 μL
JAK inhibitor I	1 μg/mL	5 μL
**Total**	**n/a**	**50 mL**

***Note:*** Store the solution at 4°C for up to one week.

**Table:** ACE medium

Reagent	Final concentration	Amount
NDiff227	n/a	∼50 mL
A83-01	1 μM	20 μL
CHIR99021	2 μM	10 μL
EGF	50 ng/mL	25 μL
**Total**	**n/a**	**50 mL**

***Note:*** Store the solution at 4°C for up to one week.

**Table:** ST medium

Reagent	Final concentration	Amount
DMEM/Ham’s F-12 (with HEPES)	n/a	∼48 mL
2ME	0.1 mM	500 μL
7.5% BSA	0.3%	10 μL
ITS-X	1%	25 μL
KSR	4%	2 mL
Forskolin	2 μM	1 μL
Y-27632	2.5 μM	12.5 μL
**Total**	**n/a**	**50 mL**

***Note:*** Store the solution at 4°C for up to one week.

**Table:** EVT-1 medium

Reagent	Final concentration	Amount
DMEM/Ham’s F-12	n/a	∼48 mL
2ME	0.1 mM	500 μL
7.5% BSA	0.3%	10 μL
ITS-X	1%	25 μL
KSR	4%	2 mL
A83-01	7.5 μM	150 μL
NRG1	100 ng/mL	50 μL
Y-27632	2.5 μM	12.5 μL
**Total**	**n/a**	**50 mL**

***Note:*** Store the solution at 4°C for up to one week.

**Table:** EVT-2 medium

Reagent	Final concentration	Amount
DMEM/Ham’s F-12	n/a	∼48 mL
2ME	0.1 mM	500 μL
7.5% BSA	0.3%	10 μL
ITS-X	1%	25 μL
KSR	4%	2 mL
A83-01	7.5 μM	150 μL
Y-27632	2.5 μM	12.5 μL
**Total**	**n/a**	**50 mL**

***Note:*** Store the solution at 4°C for up to one week.

**Table:** EVT-3 medium

Reagent	Final concentration	Amount
DMEM/Ham’s F-12	n/a	∼50 mL
2ME	0.1 mM	500 μL
7.5% BSA	0.3%	10 μL
ITS-X	1%	25 μL
A83-01	7.5 μM	150 μL
Y-27632	2.5 μM	12.5 μL
**Total**	**n/a**	**50 mL**

***Note:*** Store the solution at 4°C for up to one week.

## Step-by-step method details

### Induction of nTE

**Timing: 2–3 days**

1. Prepare Laminin-E8-coated wells beforehand.
  a. Dilute 1.5 μg (3 μL) of Laminin-E8 (iMatrix-511) in 1 mL/well of PBS and pipette the solution into one well of a newly prepared 6-well plate.
  b. Incubate at 37°C for at least 30 min.

***Note:*** The Laminin-E8 concentration is critical and is 0.15 μg/cm^2^ (0.3 μL/cm^2^). Instead of pre-coating, Laminin-E8 can be added to the medium and pipetted up and down several times after the naive PSCs are seeded in Step 5.

***Note:*** MEF feeder cells are not used for the nTE induction.

2. Dissociation of naive PSCs.
  a. Aspirate the medium from the wells and rinse with PBS two times.
  b. Add 500 μL Accutase and incubate at 37°C for 10–15 min.
  c. Add 1 mL of Wash medium and gently pipette up and down to dislodge the naive PSCs from the well.
  d. Transfer the cells to a 15-mL tube and add 5 mL of Wash medium.
  e. Centrifuge at 1,300 rpm for 3 min.
  f. Aspirate the supernatant and resuspend the pellet with 1 mL of t2iLGö medium with Y-27632.
  g. Prepare a 6-well plate and add 1 mL of 0.1% gelatin solution to a well.
  h. Aspirate the gelatin solution after 10 min of incubation at room temperature and incubate the cell suspension in 1 mL t2iLGö medium with 10 μM Y-27632 on the gelatin-coated well.
  i. Incubate at 37°C for 2 h to make the MEF feeder cells attach to the well.
  j. Collect the medium with floating PSCs into a 15-mL tube and count the number of cells.

**CRITICAL:** The quality of naive PSCs is critical. Naive PSCs show a dome-shaped morphology and express characteristic transcriptional genes such as OCT3/4, NANOG, KLF4, and KLF17. In addition, naive PSCs show specific cell surface markers such as CD75 and SUSD2. Sometimes differentiated cells can be observed as flat shaped cells. Such differentiated cells may reduce the efficiency of the subsequent nTE and nCT differentiation.

**Figure 2:**
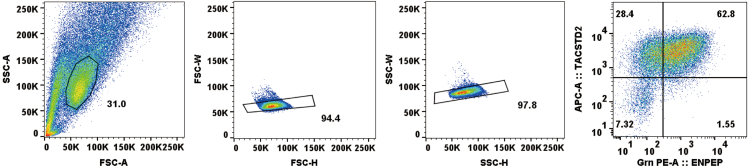
Gating strategy Flow cytometric gating strategy to sort viable TACSTD2+ENPEP+ cells from differentiated human naive pluripotent stem cells.

3. Centrifuge at 1,300 rpm for 3 min and aspirate the supernatant.
4. Resuspend with 2 mL of nTE-1 medium.
5. Seed the cells at a density of 2 **×** 10^4^–4 **×** 10^4^ cells/cm^2^ on Laminin-E8-coated dishes.
6. The following day, change the medium to nTE-2 medium.
7. Change the medium again the next day.
8. On day 3, obtain TACSTD2^+^ENPEP^+^ nTE (we obtained an efficiency of 50–60%; Figures 1B and 2).

***Optional:*** nTE can be induced without 10 ng/mL recombinant human BMP4 for the first 24 hours of the induction, although at a lower efficiency.

**CRITICAL:** The step of depriving MEF is critical. The residual MEF inhibits the differentiation of naive PSCs to nTE.

***Note:*** A higher concentration of Laminin-E8 reduces the induction efficiency of nTE. A concentration of 0.10–0.25 μg/cm^2^ Laminin-E8 is recommended.

***Note:*** hPSCs should be cultured with 10 μM Y-27632 for 24 h upon/after seeding.

***Note:*** Confirm that cell morphology on day 2 or 3 is polygonal and flat under the microscope (Figure 1B).

***Note:*** nTE strongly attaches to the dish and takes 20–30 minutes to dissociate using Accutase.

***Note:*** RNA sequencing confirmed that naive PSC-derived TACSTD2^+^ENPEP^-^ cells on day 2 and naive PSC-derived TACSTD2^+^ENPEP^+^ cells on day 3 have a high correlation with pre-implantation trophectoderm in vivo.

### Induction of nCTs

**Timing: 4 h; 3–6 days until next splitting**

nTE differentiates into nCT under ACE condition (Io et al., 2021).

9. Prepare Laminin-E8-coated wells beforehand.
  a. Add 1.5 μg (3 μL) of Laminin-E8 to 1 mL of PBS and pipette up and down several times.
  b. Add Laminin-E8/PBS solution to one well of 6-well plates.
  c. Incubate at 37°C for at least 30 min.
10. Dissociation of nTE from step 8.
  a. Aspirate the medium from the wells and rinse with PBS two times.
  b. Add 500 μL Accutase and incubate at 37°C for 20–30 min.
  c. Add 1 mL of Wash medium and gently pipette up and down to dislodge the cells from the well.
  d. Transfer the cells to a 15-mL tube and add 5 mL of Wash medium.
  e. Centrifuge at 1,300 rpm for 3 min.
11. Aspirate the supernatant and resuspend the pellet with 2 mL HBSS with 1% BSA buffer (1% BSA/HBSS) and Y-27632.
12. Incubate at 4°C for at least 30 min.
13. Centrifuge at 1,300 rpm for 3 min, aspirate the supernatant, and resuspend the pellet with 1% BSA/HBSS with anti-TACSTD2, biotin-conjugated antibody, and anti-ENPEP, PE-conjugated antibody.
14. Incubate at 4°C for 30 min in the dark.
15. Resuspend with 1 mL of 1% BSA/HBSS.
16. Centrifuge at 1,300 rpm for 3 min and aspirate the supernatant.
17. Resuspend with 1% BSA/HBSS with anti-Streptavidin, APC-conjugated antibody.
18. Incubate at 4°C for 30 min.
19. Resuspend with 1 mL of 1% BSA/HBSS.
20. Centrifuge at 1,300 rpm for 3 min and aspirate the supernatant.
21. Resuspend with 300–500 μL of 1% BSA/HBSS.
22. Sort the TACSTD2^+^ENPEP^+^ cells (Figure 2).

***Note:*** As with any cell sorting, make sure the cell sorter is clean before sorting so that contamination with other cells or microbials is avoided.

***Note:*** Keep the collection medium cool (4°C) during cell sorting to maintain the viability of the cells.

***Note:*** Cell sorting should be performed with a sufficiently rigorous selection to obtain a cell fraction of high purity (Figure 2).

23. Seed the TACSTD2^+^ENPEP^+^ cells at a density of 2 × 10^4^–4 × 10^4^ cells/cm^2^ on Laminin-E8-coated dishes in ACE medium with 10 μM Y-27632 and 1% Penicillin-Streptomycin.
24. Replace the ACE medium every two days.
25. Passage the cells every 3–6 days by dissociation with Accutase for 10–15 min and seed them at a 1:3-1:4 split ratio. Add 10 μM Y-27632 for every passage.

***Note:*** Around 0.5–1 × 10^5^ TACSTD2^+^ENPEP^+^ cells can be usually collected from 1 well of a 6-well plate by flow cytometry.

***Note:*** The combination of biotin-conjugated anti-TACSTD2 antibody and APC-conjugated anti-Streptavidin antibody demonstrate the sensitivity and resolution (Figures 1B and 2).

***Note:*** If you use fluorescent dye-conjugated antibodies as primary antibodies, you can omit the secondary antibody (anti-Streptavidin, APC-conjugated antibody).

***Note:*** If you use flow cytometry upon/after cytotrophoblast induction day 10–12, you can purify naive PSC-derived cytotrophoblasts (TACSTD2^+^ENPEP^+^SIGLEC6^+^cells) effectively.

### Maintenance of nCTs

**Timing: 4 h; 3–6 days until next splitting**

nCTs can be maintained in ACE medium for more than 40 passages as cytotrophoblast stem cells (Io et al., 2021).

**Figure 3:**
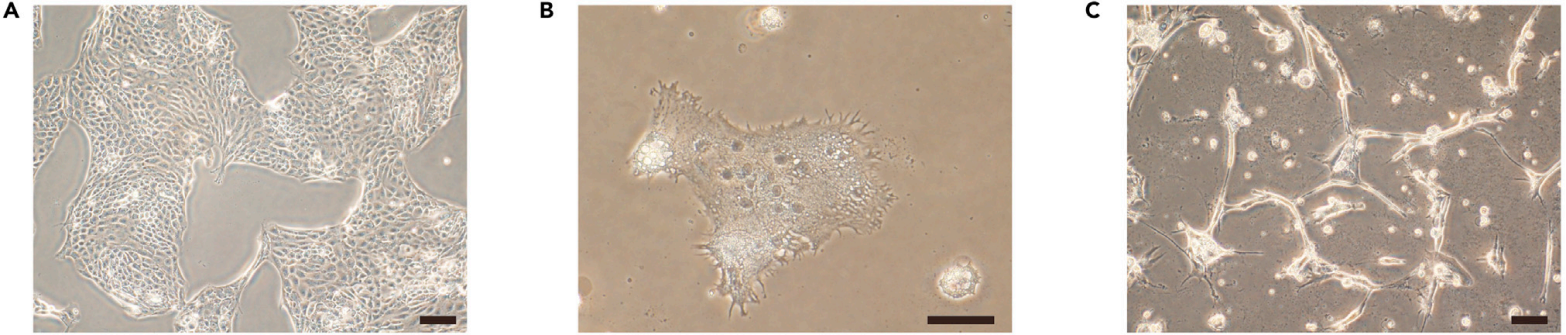
Differentiation of cytotrophoblast stem cells (A) Phase contrast image of cytotrophoblast stem cells. (B) Phase contrast image of syncytiotrophoblast derived from cytotrophoblast stem cells. (C) Phase contrast image of extravillous trophoblasts cytotrophoblast stem cells. Scale bars, 100 μm.
31. Passage the cells every 3–6 days by dissociation with Accutase for 10–15 min and seed them at a 1:3-1:4 split ratio. Add 10 μM Y-27632 for every passage.

***Note:*** nCTs should be cultured with Y-27632 for 24 h with every passage.

26. Prepare Laminin-E8-coated wells beforehand.
  a. Add 1.5 μg of Laminin-E8 to 1 mL of PBS and pipette up and down several times.
  b. Add the Laminin-E8/PBS solution to one well of 6-well dishes.
  c. Incubate at 37°C for at least 30 min in 5% O_2_.
27. Dissociation of nCTs.
  a. Aspirate the medium from the wells and rinse with PBS two times in 21% O_2_.
  b. Add 500 μL Accutase and incubate at 37°C for 10–15 min in 5% O_2_.
  c. Add 1 mL of Wash medium and gently pipette up and down to dislodge the cells from the well in 21% O_2_.
  d. Transfer the cells to a 15-mL tube and add 5 mL of Wash medium in 21% O_2_.
  e. Centrifuge at 1,300 rpm for 3 min in 21% O_2_.
28. Add ACE medium with Y-27632 for 24 h.
29. Seed the cells at a density of 4 × 10^4^ cells/cm^2^ on the Laminin-E8-coated wells in 21% O_2_ and incubate the cells in 5% O_2_, 5% CO_2_.
30. Replace the medium every two days (Figure 3A).

**Pause point:** Cells can be cryopreserved in Stem-Cellbanker and stored in liquid nitrogen.

### Differentiation into naive PSC-derived syncytiotrophoblasts (nSTs)

**Timing: 6 days**

nCTs can differentiate to nSTs according to previous protocols (Okae et al., 2018; Io et al., 2021).

32. Prepare Laminin-E8-coated wells beforehand.
  a. Add 1.5 μg (3 μL) of Laminin-E8 to 1 mL of PBS and pipette up and down several times.
  b. Add the Laminin-E8/PBS solution to one well of 6-well dishes.
  c. Incubate at 37°C for at least 30 min.
33. Dissociation of nCTs.
  a. Aspirate the medium from the wells and rinse with PBS two times.
  b. Add 500 μL Accutase and incubate at 37°C for 20–30 min.
  c. Add 1 mL of Wash medium and gently pipette up and down to dislodge the cells from the well.
  d. Transfer the cells to a 15-mL tube and add 5 mL of Wash medium.
  e. Centrifuge at 1,300 rpm for 3 min.
34. Resuspend with ST medium.
35. Seed the cells at a density of 1 × 10^4^ cells/cm^2^ on the Laminin-E8-coated wells.
36. Replace the medium at day 3.
37. Observe the nSTs on day 6 (Figure 3B).

***Note:*** The cells are incubated in 21% O_2_, 5% CO_2_ in a humidified incubator at 37°C during the induction.

***Note:*** Since ST medium contains Y-27632, no additional Y-27632 is needed at seeding.

### Differentiation into naive cell-derived extravillous trophoblasts (nEVTs)

**Timing: 8 days**

nCTs can differentiate to nEVTs according to previous protocols (Okae et al., 2018; Io et al., 2021).

38. Prepare Laminin-E8-coated wells beforehand.
  a. Add 1.5 μg (3 μL) of Laminin-E8 to 1 mL of PBS and pipette up and down several times.
  b. Add the Laminin-E8/PBS solution to one well of 6-well dishes.
  c. Incubate at 37°C for at least 30 min.
39. Dissociation of nCTs.
  a. Aspirate the medium from the wells and rinse with PBS two times.
  b. Add 500 μL Accutase and incubate at 37°C for 20–30 min.
  c. Add 1 mL of Wash medium and gently pipette up and down to dislodge the cells from the well.
  d. Transfer the cells to a 15-mL tube and add 5 mL of Wash medium.
  e. Centrifuge at 1,300 rpm for 3 min.
40. Resuspend with EVT-1 medium.
41. Seed the cells at a density of 0.8 × 10^4^ cells/cm^2^ on the Laminin-E8-coated wells.
42. Add 2% of Geltrex to the medium.
43. Replace EVT-1 medium with EVT-2 medium on day 3 and add 0.5% of Geltrex to the medium.
44. Dissociate the cells into single cells with Accutase for 10–15 min on day 6 and then resuspend with EVT-3 medium.
45. Seed the cells on new Laminin-E8-coated wells at a 1:2–1:3 split ratio.
46. Add 0.5% of Geltrex to the medium.
47. Analyze the cells on day 8 (Figure 3C).

***Note:*** The cells are incubated in 21% O_2_, 5% CO_2_ in a humidified incubator at 37°C during induction.

***Note:*** Since EVT medium contains Y-27632, no additional Y-27632 is needed at seeding.

***Optional:*** You may switch to EVT-3 medium without re-plating on day 6.

### Isolation of human cytotrophoblasts

**Timing: 6 h; 3–6 days until next splitting**

This protocol describes the use of primary patient material. Please confirm that you are allowed to process primary patient material with your local Ethics Committee and request informed consent from the donors. Placental tissues should be kept under sterile conditions.

48. Prepare Laminin-E8-coated wells beforehand.
  a. Add 1.5 μg (3 μL) of Laminin-E8 to 1 mL of PBS and pipette up and down several times.
  b. Add the Laminin-E8/PBS solution to one well of 6-well dishes.
  c. Incubate at 37°C for at least 30 min.
49. Human chorionic villi are manually separated from the chorionic membrane and decidua.
50. Cut the chorionic villi into small pieces and transfer to a 50-mL tube.
51. Rinse the villous fragments with 40 mL of sterile PBS.
52. Centrifuge at 800 rpm (120 *g*) for 2 min.
53. Aspirate most of the supernatant.
54. Repeat the rinse procedure. Shake the tube vigorously until the supernatant is clear.
55. Digest three times in a solution containing 0.25% Trypsin and 1 mg/mL collagenase IV, 200 U/mL DNase (Sigma-Aldrich), 25 mM HEPES, and DMEM/F-12 medium with agitation at 37°C.
56. Filter pooled cell suspensions through a 70-μm mesh filter (Corning) to remove debris and syncytiotrophoblasts.
57. Add 30 mL of Wash medium and centrifuge at 1,300 rpm for 3 min.
58. Resuspend with 1% BSA/HBSS and incubate on ice for 30 min to reduce the non-specific binding of antibodies.
59. Centrifuge at 1,300 rpm for 3 min, aspirate the supernatant, and resuspend the pellet with 1% BSA/HBSS with an Alexa Fluor 488-conjugated anti-TACSTD2 antibody, a PE-conjugated anti-ENPEP antibody, and a biotin-conjugated, anti-SIGLEC6 antibody.
60. Incubate at 4°C for 30 min.
61. Resuspend with 1 mL of 1% BSA/HBSS.
62. Centrifuge at 1,300 rpm for 3 min and aspirate the supernatant.
63. Resuspend with 1% BSA/HBSS with anti-Streptavidin, APC-conjugated antibody.
64. Incubate at 4°C for 30 min.
65. Collect TACSTD2^+^ENPEP^+^SIGLEC6^+^ cells using a cell sorter.

***Note:*** Keep the collection medium cool (4°C) during cell sorting to maintain the viability of the cells.

***Note:*** Cell sorting should be performed with a sufficiently rigorous selection to obtain high-purity cell fraction.

***Note:*** Given the increased risk of microbial contamination during purification step by flow cytometry, collected cells may be fed with ACE medium supplemented with 1% Penicillin-Streptomycin. Antimicrobial-supplemented medium should be used for a minimum of 2 days.

66. Centrifuge at 1,300 rpm for 3 min.
67. Aspirate the supernatant and resuspend with ACE medium supplemented with 1% Penicillin-Streptomycin.
68. Seed the cells at a density of 4 × 10^4^ cells/cm^2^ on the Laminin-E8-coated wells.
69. Replace the medium every two days. The cells are passaged every 3–6 days by dissociation with Accutase for 10–15 min and seeded at a 1:3–1:4 split ratio. 10 μM Y-27632 is added with every passage.

***Note:*** If you use fluorescent dye-conjugated antibodies as the primary antibodies, you can omit the secondary antibody reaction.

***Note:*** Pacific Blue-conjugated HLA-ABC antibody improves the purity of cytotrophoblasts (TACSTD2^+^ENPEP^+^SIGLEC6^+^HLA-ABC^-^ cells).

**CRITICAL:** To maintain human primary cytotrophoblasts, cells should be sorted from first-trimester placenta. Cytotrophoblasts sorted from full-term placenta cannot be maintained with ACE medium.

## Expected outcomes

We can purify trophectoderm-like cells from naive PSCs.

nTE expresses trophectoderm markers, such as CDX2, GATA3, TFAP2C and KRT19, as well as HAVCR1, ITGA6, and SLC12A3.

nCTs maintain trophoblast markers and have the capacity to differentiate to nSTs and nEVTs according to previous reports (Okae et al., 2018; Io et al., 2021) (Figure 3).

Primary human cytotrophoblasts can be maintained and differentiated to STs and EVTs by the same procedure used for nCTs.

## Limitations

The induction efficiency of nTE is highly dependent on the quality of the naive PSCs. We recommend that you check the quality of your naive PSC cultures before they are used for the experiments. It is also important to use several cell lines.

Although the global gene expression profiles of nTE and nCTs are very similar to their in vivo counterparts, the epigenomes of nTE and nCTs were not evaluated because there is less epigenetic data of in vivo human trophoblast development.

We usually obtain in vivo samples of human trophoblasts at 5–7 weeks pregnancy or full-term pregnancy. We do not know if cytotrophoblast stem cells can be established at other stages. Since we cannot access E14-E21 human embryos, we do not have in vivo human data on this stage.

## Troubleshooting

### Problem 1

Low efficiency of nTE induction (steps 1–7).

### Potential solution

The Laminin-E8 concentration affects the efficiency. The recommended concentration is 0.10–0.25 μg/cm^2^. Another possible cause is the contamination of MEF feeder cells during the nTE induction. If fibroblasts are observed in addition to naive cell-derived cells on the day after the nTE induction, less nTE is induced. In this case, MEF feeder cells should be removed more strictly. Longer incubation times of dissociated cells under gelatin-coated dishes will help remove MEF feeder cells, but also less PSCs are harvested. Repeat the procedure several times to determine the ideal incubation time.

### Problem 2

Many differentiated cells are mixed with naive cells (steps 2).

### Potential solution

Differentiated cells may reduce the induction efficiency of nTE or nCTs. The longer naive cells are cultured, the more likely differentiated cells are to appear. When MEFs are removed in Step 2i, the differentiated cells also stick to the bottom of the dish to some extent and are removed. However, if there are many differentiated cells and the induction rate is low, it is better to establish new naive cells from primed cells.

### Problem 3

Excessive cell death during nTE induction (steps 5–7).

### Potential solution

Adding Y-27632 to cultured naive PSCs at least 30 min before the dissociation can prevent cell death during the induction.

### Problem 4

Excessive cell death during nCTs induction (steps 23–25).

### Potential solution

Adding Y-27632 to cultured nTE at least 30 min before the dissociation can prevent cell death during the induction. Immunostaining and cell sorting should be done as quickly as possible to avoid cell damage.

### Problem 5

Excessive loss of cells during the digestion procedure of human chorionic villi (steps 55–57).

### Potential solution

If the amount of chorionic villi specimen is too high compared with the digestive solution, cell clumps sometimes appear, and the single-cell isolation does not work well. Using a concentrated digestive solution, the samples can be dissociated completely without sticky clumps. DNase also reduces the formation of sticky cell clumps.

### Problem 6

Many red blood cells are mixed in the sample (steps 55–57)

### Potential solution

The most important and simple solution is to wash with PBS repeatedly. Additionally, you can use red blood cell lysis buffer (Santa Cruz Biotechnology Cat. sc-296258) to remove red blood cells. Density gradient reagents like Debris Removal Solution (Miltenyi Biotec Cat. 130-109-398) also may remove red blood cells.

## Resource availability

### Lead contact

Further information and requests for resources and reagents should be directed to and will be fulfilled by the lead contact, Yasuhiro Takashima (y.takashima@cira.kyoto-u.ac.jp).

### Materials availability

This study did not generate new unique reagents.

## Data Availability

This study did not generate datasets and codes.
